# Re: Intracranial primary collision tumours: A comprehensive systematic review on preoperative radiological accuracy and neuro-oncological insights. Brain Spine 2026: 105943

**DOI:** 10.1016/j.bas.2026.105950

**Published:** 2026-01-26

**Authors:** Tayfun Hakan

**Affiliations:** a University of Health Sciences, Hamidiye Faculty of Medicine, Department of Surgical Medical Sciences, Department of Neurosurgery, İstanbul, Turkey; b İstanbul Fatih Sultan Mehmet Training and Research Hospital, İstanbul, Turkey

**Keywords:** Collision tumours, Glioblastoma, Meningioma, Neuroimaging, Neurooncology

Dear Editor,

I read with great interest the recent article published in Brain and Spine by Jadoon et al. (2026), in which the authors provide a comprehensive and well-structured review of intracranial primary collision tumours. The authors are to be congratulated for addressing this rare and diagnostically challenging entity, which continues to raise important pathogenetic and conceptual questions in neuro-oncology.

I was pleased to note that one of my earlier publications was cited in this review (Hakan et al., 1998). This citation offers an opportunity to briefly revisit the historical context of that report and to reflect on its relevance in light of contemporary advances in molecular neuro-oncology.

At the time our study was conducted, intracranial collision tumours were generally regarded as coincidental findings. Their etiology remained largely speculative, with hypotheses including shared environmental exposure, local tissue susceptibility, or an underlying predisposition of the surrounding brain parenchyma. Importantly, comprehensive molecular and genetic analyses were not feasible under the technical and infrastructural conditions of that period. As a result, interpretations were necessarily based on clinical presentation, radiological findings, intraoperative observations, and conventional histopathological examination.

Despite these limitations, our report contributed to the early recognition that the coexistence of two histologically distinct primary brain tumours within the same anatomical region might not be entirely incidental. This question remains highly relevant today. Even with the significant progress achieved in molecular neuro-oncology, the precise mechanisms underlying intracranial primary collision tumours have not yet been fully elucidated. Their rarity and biological heterogeneity continue to limit systematic investigation and the development of unified pathogenetic models.

Recent advances in molecular profiling have substantially reshaped our understanding of tumour development and coexistence. Techniques such as whole-exome sequencing, DNA methylation profiling, and copy number variation analysis have demonstrated that spatially adjacent or synchronous tumours may harbor distinct molecular landscapes and independent driver events. In meningiomas in particular, accumulating molecular evidence has highlighted substantial biological heterogeneity and distinct molecular pathways in sporadic, non-NF2-associated tumours (Clark et al., 2013).

While hereditary conditions such as neurofibromatosis type 2 provide a well-established framework for the development of multiple meningiomas, the majority of reported cases of intracranial tumour coexistence—including collision tumours—occur in patients without clinical or genetic evidence of NF2. In such sporadic cases, increasing recognition of molecular and biological heterogeneity among lesions suggests that these tumours may arise through distinct pathogenetic mechanisms. This observation raises the possibility that local microenvironmental influences, regional susceptibility, or patient-specific factors may contribute to the synchronous or metachronous development of distinct neoplasms.

In this context, I would like to draw attention to an additional observation related to intracranial tumour coexistence. In a case published on the EuroRad platform, we reported the presence of two meningiomas with different histological characteristics within the same cerebral hemisphere (Hakan et al., 2001). Although this case does not represent a classic collision tumour involving two distinct tumour entities, it nevertheless illustrates the biological heterogeneity that may exist within a single intracranial compartment. Such findings further support the notion that multiple, histologically distinct neoplasms can develop independently within the same patient and anatomical region.

At the time of its publication, this case was disseminated through an online radiological repository rather than a widely indexed journal. Given the relatively limited visibility of such platforms—particularly during earlier phases of internet-based scientific dissemination—it is possible that this observation did not receive the broader attention it might otherwise have achieved. Nevertheless, it represents an early contribution to the ongoing discussion regarding tumour coexistence, heterogeneity, and independent tumorigenesis within the cranial compartment.

Revisiting and contextualizing such early observations is valuable both for historical completeness and for informing future research. Case reports published prior to the molecular era, although constrained by the methodologies available at the time, may acquire renewed significance when interpreted alongside contemporary genetic and epigenetic data. Increased awareness of these observations may help bridge the gap between classical clinicopathological descriptions and modern pathogenetic concepts.

In conclusion, the etiology of intracranial primary collision tumours remains incompletely understood. While genetic and molecular mechanisms are increasingly implicated, comprehensive pathogenetic models have yet to be fully established. It is hoped that ongoing advances in molecular diagnostics will allow longstanding questions to be addressed more conclusively and that early clinical observations—some of which may have been underrecognized—will continue to enrich and inform current research.

I thank the authors for their valuable contribution to this evolving field and hope that this correspondence adds constructively to the ongoing discussion.

Tayfun Hakan.

## Funding sources

This research did not receive any specific grant from funding agencies in the public, commercial, or not-for-profit sectors.

## Conflict of interest

The authors declare that they have no known competing financial interests or personal relationships that could have appeared to influence the work reported in this paper.
